# Tomato Cultivars With Variable Tolerances to Water Deficit Differentially Modulate the Composition and Interaction Patterns of Their Rhizosphere Microbial Communities

**DOI:** 10.3389/fpls.2021.688533

**Published:** 2021-07-13

**Authors:** Alexis Gaete, Rodrigo Pulgar, Christian Hodar, Jonathan Maldonado, Leonardo Pavez, Denisse Zamorano, Claudio Pastenes, Mauricio González, Nicolás Franck, Dinka Mandakovic

**Affiliations:** ^1^Laboratorio de Bioinformática y Expresión Génica, Instituto de Nutrición y Tecnología de los Alimentos, Universidad de Chile, Santiago, Chile; ^2^Center for Genome Regulation, Santiago, Chile; ^3^Programa de Doctorado en Ciencias Silvoagropecuarias y Veterinarias, Campus Sur Universidad de Chile, Santiago, Chile; ^4^Laboratorio de Genómica y Genética de Interacciones Biológicas (LG^2^IB), Instituto de Nutrición y Tecnología de los Alimentos, Universidad de Chile, Santiago, Chile; ^5^Laboratorio de Biología de Sistemas de Plantas, Departamento Genética Molecular y Microbiología, Facultad de Ciencias Biológicas, Pontificia Universidad Católica de Chile, Santiago, Chile; ^6^Instituto de Ciencias Naturales, Universidad de Las Américas, Santiago, Chile; ^7^Departamento de Ciencias Químicas y Biológicas, Universidad Bernardo O’Higgins, Santiago, Chile; ^8^Centro de Estudios en Zonas Áridas (CEZA), Universidad de Chile, Coquimbo, Chile; ^9^Facultad de Ciencias Agronómicas, Universidad de Chile, Santiago, Chile; ^10^GEMA Center for Genomics, Ecology and Environment, Universidad Mayor, Santiago, Chile

**Keywords:** plant tolerance, water deficit, *Solanum lycopersicum* cultivars, rhizosphere microbial community, network interactions

## Abstract

Since drought is the leading environmental factor limiting crop productivity, and plants have a significant impact in defining the assembly of plant-specific microbial communities associated with roots, we aimed to determine the effect of thoroughly selected water deficit tolerant and susceptible *Solanum lycopersicum* cultivars on their rhizosphere microbiome and compared their response with plant-free soil microbial communities. We identified a total of 4,248 bacterial and 276 fungal different operational taxonomic units (OTUs) in soils by massive sequencing. We observed that tomato cultivars significantly affected the alpha and beta diversity of their bacterial rhizosphere communities but not their fungal communities compared with bulk soils (BSs), showing a plant effect exclusively on the bacterial soil community. Also, an increase in alpha diversity in response to water deficit of both bacteria and fungi was observed in the susceptible rhizosphere (SRz) but not in the tolerant rhizosphere (TRz) cultivar, implying a buffering effect of the tolerant cultivar on its rhizosphere microbial communities. Even though water deficit did not affect the microbial diversity of the tolerant cultivar, the interaction network analysis revealed that the TRz microbiota displayed the smallest and least complex soil network in response to water deficit with the least number of connected components, nodes, and edges. This reduction of the TRz network also correlated with a more efficient community, reflected in increased cooperation within kingdoms. Furthermore, we identified some specific bacteria and fungi in the TRz in response to water deficit, which, given that they belong to taxa with known beneficial characteristics for plants, could be contributing to the tolerant phenotype, highlighting the metabolic bidirectionality of the holobiont system. Future assays involving characterization of root exudates and exchange of rhizospheres between drought-tolerant and susceptible cultivars could determine the effect of specific metabolites on the microbiome community and may elucidate their functional contribution to the tolerance of plants to water deficit.

## Introduction

One of the significant consequences of climate change will be an increased frequency and severity of drought (Vrochidou et al., 2013; Leng et al., 2015), which is a major complication considering that water scarcity is the most adverse environmental factor limiting crop productivity (Comas et al., 2013). A fruit crop that would be highly affected by this abiotic stress is tomatoes, given that, under drought, its fruit yield is significantly reduced (Foolad et al., 2003; Nuruddin et al., 2003; Landi et al., 2016; Qi et al., 2016). This is quite relevant considering that this horticultural species is a major component of diet, with almost six million hectares planted worldwide and over 200 million tons produced yearly (FAOSTAT, 2017). Therefore, the study of the relationships that tomatoes establish with their environment in the context of adaptation to low water availability becomes an essential task for sustainable agricultural development.

Plants undergo a series of responses to drought to protect themselves from its damaging effects. These responses include morphological, physiological, biochemical, and metabolic alterations in all plant tissues (Cochard et al., 2002). Drought tolerance has been related to an improvement in the water-use efficiency by decreasing stomatal conductance and transpiration rate to maintain a higher photosynthetic rate (Li et al., 2017), ensuring the correct use of the plant’s available water to be safe and to maintain its productivity level (Mega et al., 2019).

The quantity and composition of root exudates are determined by plant genotypes (Badri and Vivanco, 2009; Gargallo-Garriga et al., 2018). Consequently, since root exudates have been described as main drivers in shaping rhizosphere microbial communities (Badri and Vivanco, 2009; Dennis et al., 2010; Shi et al., 2011; Bulgarelli et al., 2013; Sasse et al., 2018), the host has a significant impact in defining the assembly of plant-specific microbial communities associated to the roots (Berg and Smalla, 2009; Bouffaud et al., 2014; Ofek-Lalzar et al., 2014; Bulgarelli et al., 2015; Li et al., 2018). This host-specific community configuration is crucial under the “holobiont” concept, which considers the multicellular host and its associated microbiota as a functional entity with a bidirectional relationship (Rosenberg and Zilber-Rosenberg, 2016). In the holobiont, the root zone is heavily enriched in compounds that are secreted by both plants and microorganisms and play a key role in maintaining plant-microbe interactions (Badri and Vivanco, 2009). Plant root exudates provide photosynthate carbon for microbial growth and facilitate direct communication between plants and microbes *via* signaling molecules and phytohormones (De Vries et al., 2020). On the other hand, rhizosphere microorganisms contribute to major functions, such as plant nutrition and plant resistance to biotic and abiotic stresses (Vandenkoornhuyse et al., 2015; Garcia and Kao-Kniffin, 2018). Thus, the plant microbiome appears to be part of the significant portion of missing adjustments attributable to the environment (Chapman et al., 1997; Xu, 2016) that may explain plant tolerance to unfavorable environmental conditions (Turner et al., 2013; Naylor and Coleman-Derr, 2018).

Ongoing research is ample in information about the abilities of specific soil microbial strains to influence drought tolerance in plants (Niu et al., 2018; Meenakshi et al., 2019; Chukwuneme et al., 2020). Nonetheless, these studies only provide a snapshot of the complex interactions that occur in the rhizosphere, given that in nature, plants not only interact with beneficial microorganisms but also and simultaneously with detrimental microbes (Philippot et al., 2013; Schlaeppi and Bulgarelli, 2015). Thus, researchers are left with a limited understanding of the role of the broader root microbiome on the ability of a plant to overcome abiotic stresses, such as drought (Garcia and Kao-Kniffin, 2018).

This study aimed to systematically select tomato (*Solanum lycopersicum*) cultivars tolerant and susceptible to water deficit to evaluate microbiomes of their rhizospheres. By doing so, we evaluated their taxonomic bacterial and fungal composition and interaction patterns to determine the effect of the plant cultivar on the microbiome response to water deficit. Additionally, to assess the effect of plants on the soil microbial response to water deficit, we also sequenced the microbial communities of bulk soils under this adverse condition. Here, we provide new and profound information on the influence of differential adaptability of plants to water deficit on its surrounding microbiota.

## Materials and Methods

### Assay Design and Plant Physiological Measurements

Three cultivation assays were performed to select the most and less tolerant to water scarcity cultivars for posterior microbiome analyses. A scheme of the assay design is displayed in Supplementary Figure 1.

First selection assay. Seventy-two *S. lycopersicum* cultivars available from La Platina Genetic Resources Unit and Germplasm Bank (Instituto de Investigaciones Agropecuarias de Chile, INIA, Chile) (*n* = 10 for each of the 72 cultivars; 720 plants in total) were cultivated in a complete random design in December 2012 from sterilized seeds at Centro Regional de Investigación La Platina from Instituto de Investigaciones Agropecuarias de Chile (INIA) located in Santiago, Chile, in a field experiment. Plants were maintained under full irrigation (FI) for 75 days three times a week, keeping the soil between field capacity and 80% of field capacity. Later, irrigation was suppressed for all plants (withholding irrigation; WI) for 21 days. According to a visual score (based on turgor of leaves and plants), the extent of wilting was used to select the 10 most tolerant and the 10 most susceptible cultivars to drought.

Second selection assay. The twenty cultivars selected in the first selection assay (*n* = 6 for each of the 20 cultivars; 120 plants in total) were cultivated from sterilized seeds for 75 days in FI and 21 days in FI (*n* = 3) or WI (*n* = 3), in 20 L pots in a greenhouse trial. This assay was performed at the Centro Regional de Investigación La Platina from Instituto de Investigaciones Agropecuarias de Chile (INIA) located in Santiago, Chile. Commercial topsoil was used as the soil substrate for this assay. The greenhouse was maintained at 16 h/8 h photoperiod, 25°C/15°C (day/night), and relative humidity of 50 ± 10% for the trial duration. Plants were cultivated in a completely random design inside the greenhouse. At the end of the assay, the leaf relative water content (RWC) from all plants was determined by weighing the leaf fresh weight, leaf dry weight, and saturated weight after 18 h floating of leaves on water using the formula described by Anjum et al. (2016). The dry weight of the leaves was obtained by oven drying at 70°C for 72 h. This measurement allowed the selection at the end of this assay of the three most tolerant and the three most susceptible cultivars to drought.

Third selection assay. The six cultivars selected in the second selection assay (*n* = 6 for each of the six cultivars; 36 plants in total) were cultivated in a greenhouse (without photoperiod, temperature, or relative humidity regulation) trial located at Experimental Station “Las Cardas,” Facultad de Ciencias Agronómicas, Universidad de Chile, in the Limarí Valley, Coquimbo, Chile. The local semi-arid soil was used as a substrate for plant cultivation, which is of sandy loam texture and originated from marine sediments of the “tambillo” series (typic haloduric) with less than 1% organic matter. Mean temperature and relative humidity inside the greenhouse during the assay were 22.8 ± 2.4°C and 52.3 ± 4.6%, respectively and were recorded through Vantage Pro2 Weather Station (Davis Instruments, Hayward, CA, United States). Sterilized seeds were used, and plants (in 20 L pots) were maintained at field capacity, irrigating three times a week (full irrigation; FI) for 120 days. Also, pots filled with soil but without plants (“bulk soil” or BS; *n* = 6) were maintained under the same conditions of the plants throughout the assay. Pots were distributed in a completely random design inside the greenhouse, distanced by 50 cm between rows. At the initial time (Ti), considered 120 days after seeding and before treatment began, three replicates of each cultivar and three BS replicates were maintained in FI for 21 days, while the other three replicates were submitted to 21 days of deficit irrigation (DI). DI consisted of 50% of FI for 14 days, after which irrigation was withheld, exposing the plants to a progressive drought for seven days. In this assay, RWC, photosynthesis (Pn), and stem water potential (Ψ_stem_) were measured in all plants at Ti and at the end of the assay (final time; Tf). RWC was measured as described before. Pn was assessed by employing an open portable photosynthesis system Li-6400 (LI-COR Biosciences, Lincoln, NE, United States) equipped with a LI−6400−40 Leaf Chamber Fluorometer. Measurements were performed on fully sun−exposed and extended leaves. During these measurements, air CO_2_ concentration was controlled using the injection system and compressed CO_2_ cylinders with a CO_2_ concentration of 380 ppm, and were done at a saturating PAR light intensity, between 11:00 and 13:00, which is the period of maximum photosynthetic rates. The area of the leaves was measured to correct the LICOR outputs. Ψ_stem_ measurements were performed in adult mature and fully expanded non-transpiring leaves directly connected to the main stem, which had been bagged with both plastic sheet and aluminum foil at least 1 h before measurement (Choné et al., 2001). Bagging prevented leaf transpiration, so leaf water potential equaled stem water potential (Begg and Turner, 1970). Each bagged leaf was then cut from the plant and placed inside a pressure chamber (model 1505D, PMS Instrument, OR, United States) for measurement.

All trials were daily inspected, and pests and diseases were controlled promptly, while weeds were controlled manually as soon as they were detected.

Shoot areas were measured in all plants under FI and DI at Tf through digitalized images. The shoot area corresponded to the sum of areas of individual leaf per plant. Images of the leaves were obtained using a common personal computer scanner, in JPEG format, and a 200 dpi resolution. In each scan, a 1-cm^2^ portion of leaf was included as a known reference area. Both the leaf area and reference area were individually counted in pixels through the magic wand tool of the Adobe Photoshop CS2 software according to the image processing methodology proposed by Sandrini-Neto et al. (2007). Subsequently, to obtain the leaf area (cm^2^), the number of pixels of each leaf was multiplied by the reference area and divided into the reference number of pixels. Root areas were also calculated in all plants under FI and DI at Tf. The root images were prepared spreading the roots in a transparent glass tray, also containing a 3-mm water layer according to the Costa et al. (2014) methodology, and were obtained using a common personal computer scanner (optical resolution 1,200 dpi × 1,200 dpi). Root surface was calculated using the ImageJ software version 1.50i. The images were linearized to bring the roots to the foreground.

### Rhizosphere Sampling, DNA Extraction and Sequencing

Rhizospheres from the most susceptible and tolerant cultivars determined at the end of the third selection assay (triplicates for FI and DI treatments) were obtained by gently shaking the root system to dislodge small adhering soil clumps. Roots were cut and washed in a 10-mM NaCl solution (Gottel et al., 2011; Bulgarelli et al., 2012), and the soil detached (rhizosphere) was collected in 50-ml tubes (Fernández-Gómez et al., 2019). For sampling BS, around 100 g of soil from pots that were never in contact with plants (triplicates for FI and DI treatments) were obtained at 15 cm depth in sterile plastic bags. All soils were immediately frozen (−20°C) until DNA extraction.

DNA was extracted from each of the soil samples using the DNeasy Blood and Tissue kit (Qiagen, Germany) combined with the Cetyl trimethylammonium bromide (CTAB) based method (Zhou et al., 1996; Prestel et al., 2008). Briefly, 5 g of soil were resuspended in a 5-ml extraction buffer [100 mM Tris–HCl, pH 8; 100 mM Na EDTA, pH 8; 100 mM Na_2_HPO_4_, 1.5 M NaCl, 1% (w/v) CTAB], and then 10mg/ml (final concentration) of lysozyme and 10 μl of proteinase K (20 mg/ml) were added and mixed by vortex, followed by incubation at 65°C for 2h with constant mixing. The mixture was centrifuged at 2,000 × *g* for 5 min at room temperature, and the supernatant fluid was transferred to a clean tube and mixed by vortex with 1 ml of AL Binding Buffer (Qiagen, Germany) and 1 ml of ethanol 100%. The mixture was transferred into the DNeasy mini spin column to continue the kit protocol. DNA integrity was evaluated by electrophoresis in Agilent 2200 TapeStation (Agilent Technologies, Santa Clara, CA, United States), and only good quality DNA was selected for sequencing. DNA concentration was measured by fluorescence using the Qubit dsDNA HS Assay Kit (Thermo-Fisher Scientific, Waltham, MA, United States). Then, DNA was stored at 4°C until it was used.

Microbial DNA was amplified using a bacteria-specific primer set, 28F (5′-GA GTT TGA TCM TGG CTC AG-3′) and 519R (5′-GWA TTA CCG CGG CKG CTG-3′), flanking variable regions V1–V3 of the 16S rRNA gene (Turner et al., 1999), and ITS1 (5′-TCC GTA GGT GAA CCT GCGG-3′) and ITS2 (5′-GCT GCG TTC TTC ATC GAT GC-3′), flanking yeast ITS1 variable region (White et al., 1990), in both cases with a barcode on the forward primer. Amplification was performed using the HotStarTaq Plus Master Mix (Qiagen, Germany) under the following conditions: initial denaturation at 94°C for 3 min followed by 28 cycles, each set at 94°C for 30 s, 53°C for 40 s, and 72°C for 1 min, with a final elongation step at 72°C for 5 min. PCR products were used to prepare DNA libraries following the Illumina TruSeq DNA library preparation protocol. Sequencings were performed at the Molecular Research DNA laboratory (Shallowater, TX, United States) on an Illumina MiSeq platform (Illumina, San Diego, CA, United States) overlapping 2 bp × 300 bp configuration with a minimum throughput of 40,000 reads per sample.

### Processing of Illumina Sequence Data

Raw amplicon sequences were processed and analyzed following previously described protocols (Dowd et al., 2008; Handl et al., 2011). Reads were processed in Mothur v.1.42.1 (Schloss et al., 2009) with default parameters. Briefly, sequences were joined (overlapping pairs) and grouped by samples following the barcodes, and then the barcodes were removed. Then, sequences < 150 bp or with ambiguous base calls were removed. The remaining sequences were filtered using the USEARCH clustering algorithm at 4% sequence divergence to remove chimeras and clusters consisting of only one sequence (i.e., singletons) (Edgar, 2010; Edgar et al., 2011). Finally, sequences were quality filtered with Mothur v.1.42.1 (Schloss et al., 2009) with the minimal quality average set to 30.

### Taxonomic Identification

Sequences were analyzed with the software Quantitative Insights Into Microbial Ecology (QIIME v1.8.0) (Caporaso et al., 2010). Briefly, we used QIIME script “pick_closed_reference_otus.py” to extract all 16S rRNA or ITS reads from the amplicon data that matched the SILVA r16S database (version 138) (Quast et al., 2013) or the Unite Community ITS database (version 7.2) (Nilsson et al., 2019), respectively, at 97% of similarity or 3% divergence, with the taxonomy of the resulting OTUs assigned directly from the closest sequence match (“mapped reads”). The OTU picking process was performed with USearch v6.1.544 (Edgar, 2010; Edgar et al., 2011) using QIIME default parameter values (−s.97 –z True–max_accepts 1–max_rejects 8–word_length 8–minlen 64–usearch61_sort_method abundance). Singletons were removed. Sequences other than bacteria and fungi were removed. For analyses, we selected reads that mapped with OTUs that were identified in at least two out of the three samples of each soil type and water irrigation treatment to analyze data using representative OTUs of each soil/treatment combination.

### Microbial Diversity Analysis

To characterize microbial diversity metrics, alpha OTU diversity by random subsampling (without replacement) was calculated for each soil sample using the alpha_rarefaction.py script in QIIME. The observed number of species (Richness) and Shannon index H were calculated. Rarefaction curves were obtained for each of these metrics by serial subsampling in increments of 3,900 sequences and 10 iterations per increment, to a standardized 39,000 sequences per sample for 16S rRNA samples, and serial subsampling in increments of 1,400 sequences and 10 iterations per increment, to a standardized 14,000 sequences per sample for ITS samples. The 10 sequence subgroups of the final serial subsampling step of each sample were used to compare the observed number of species and Shannon index between the soil type/water irrigation treatments. Beta diversity was evaluated by principal coordinates analysis (PCoA) and unweighted pair group method with arithmetic mean (UPGMA) tree with Bray–Curtis distance at phylum and family taxonomic levels as defined by the software MEGAN Community Edition v6.19.9 (Huson et al., 2016).

### Microbial Interaction Networks in Response to Water Deficit

To generate microbial interaction networks from the soil samples, we first selected reads that mapped with OTUs identified in at least four out of the six samples from each soil. Also, the OTU relative abundance ratio of deficit irrigated over full irrigated plants was used instead of OTU relative abundances of every soil compartment to recover in the network, only the ecological interactions established entirely from the water deficit treatment. For this, samples with zero (0) relative abundance were replaced by a one (1) since any number divided by one is equal to the number itself; therefore, the abundance difference between DI and FI was maintained. Hence, the input data were the nine abundance DI/FI ratios obtained for each OTU (three DI replicates and three FI replicates), always maintaining the order of the samples. The outputs were the “response-to-water-deficit” interaction networks from each soil compartment. Briefly, significant positive or negative co-responses to water deficit across the samples were identified by the CoNetCytoscape plug-in method (Faust and Raes, 2016). They were inferred according to the ratio abundance correlation pattern of pairs of OTUs over the samples using a measure that quantifies their ratio distribution similarity. When two OTUs showed a similar ratio abundance pattern over the samples, a positive co-response to water deficit was acknowledged. When they presented an anticorrelation in their ratio abundance pattern, a negative co-response to water deficit was accepted. After assessing all possible combinations of OTUs in the ratio abundance data set, all significant pairwise relationships were combined to construct the network (Faust and Raes, 2012) using a multiple ensemble correlation. Four similarity measures were calculated: Bray Curtis and Kullback-Leibler non-parametric dissimilarity indices; Pearson and Spearman rank correlations. For each measure and each edge, 1,000 renormalized permutation and bootstrap scores were generated according to Faust et al. (2012). “Responsive-to-water-deficit hub nodes” were considered 1% nodes that presented the highest degrees in each network. In the case of the BS network, more than 1% of the nodes presented the same highest connectivity (59 nodes with a degree over 30); thus, they were all considered responder hub nodes. Interaction network models were displayed by Cytoscape (Shannon et al., 2003), which revealed the parameters of the networks.

### Statistical Analyses

Shapiro–Wilk normality test (*p* < 0.05) was performed to check the distribution of the samples. Plant physiological measurements from the second and the third selection assays of the cultivars were compared by Student’s *t*-test (*p* < 0.05). Microbial diversity (Richness and Shannon index H) comparisons among conditions were evaluated by Kruskal–Wallis test and Dunn’s *post hoc* test (*p* < 0.05). Microbial taxonomic differences between irrigation treatments in each soil sample were compared by Student’s *t*-test (*p* < 0.05). The enrichment analysis of the taxa in the microbial interaction networks compared with the total community was calculated based on the cumulative hypergeometric distribution (*p* < 0.01). All the analyses were done using the GraphPad Prism 5 software.

## Results

### Selection of Tolerant and Susceptible *Solanum lycopersicum* Cultivars

**FIGURE 1:**
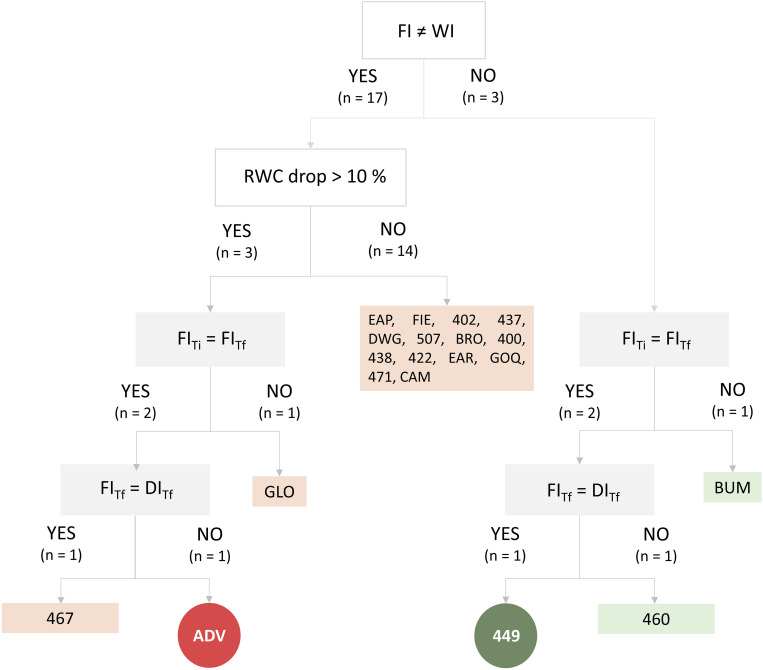
Classification tree (Rokach and Oded, 2015; Xue et al., 2015) based on plant physiological parameters to evaluate which cultivars were more tolerant and susceptible to deficit irrigation. White boxes represent conditionals evaluated in the second selection assay (RWC drop > 10%); gray boxes represent conditionals evaluated in the third selection assay (RWC, Pn, and Ψ_stem_). Edges represent the answers (yes or no) to the conditionals. Conditional output codes correspond to IDs of cultivars (Supplementary Table 1). Light green (boxes) conditional outputs represent tolerant cultivars; pink (boxes) conditional outputs represent susceptible cultivars. Circular dark green conditional output (449) represents the most tolerant cultivar; circular dark red conditional output (ADV) represents the most susceptible cultivar. FI, full irrigation treatment; WI, withholding irrigation treatment; DI, deficit irrigation treatment. Ti and Tf represent the initial and final times of the assay. RWC, relative water content.

Three selection assays were performed to identify the most tolerant and the most susceptible *S. lycopersicum* cultivars to water deficit. The first selection assay permitted the assortment from 72 *S. lycopersicum* cultivars of the 10 most tolerant and 10 most susceptible cultivars to drought by visual wilting. These 20 cultivars were categorized in a classification tree (Figure 1), which included conditionals from a second and a third selection assay. In the second selection assay, we evaluated if the leaf RWC of the plant was significantly different between FI and WI treatments for each of these 20 cultivars. The results indicated that three cultivars revealed no significant differences between FI and WI (BUM, 460, and 449), while the other three cultivars exhibited significant decreases (greater than 10%) in WI compared with FI (GLO, ADV, and 467); hence, were categorized as “tolerant” and “susceptible,” respectively. Afterward, in a third assay, we tested if the susceptible/tolerant phenotype manifested was recapitulated in an arid zone soil under FI and deficit irrigation (DI) conditions by measuring three physiological parameters (RWC, Pn, and Ψ_stem_). Under FI conditions, the RWC of GLO and BUM changed significantly between the Ti and the Tf of the assay; hence, these cultivars were discarded since their differences were time-dependent and not necessary water deficit-dependent. Among the remaining cultivars, ADV was the only one that exhibited significantly lower RWC, Pn, and Ψ_stem_ values in DI compared with FI; therefore, it was selected as the cultivar most susceptible to water deficit. On the contrary, only the 449 cultivar was considered tolerant to water deficit, because it did not display differences between FI and DI for the three variables examined. The complete dataset of these analyses is shown in Supplementary Table 1.

Regarding morphological differences of the plants, neither ADV nor 449 exhibited significant differences in their plant shoots between FI and DI treatments. However, a significant decrease in the root area of the susceptible cultivar was observed in DI compared with FI (Supplementary Figure 2).

### Taxonomic Composition of Rhizosphere Microbial Communities From Tolerant and Susceptible Cultivars

In order to compare the rhizosphere microbiome associated with ADV (susceptible cultivar rhizosphere; SRz) and 449 (tolerant cultivar rhizosphere; TRz) in response to water deficit, we sequenced the entire bacterial and fungal communities by the high-throughput metabarcoding sequencing (HTS) technology, under FI and DI treatments. Also, to evaluate the plant effect in response to water deficit, BS microbiomes were sequenced under both irrigation treatments. A total of 2,697,284 16S rRNA bacterial and 889,016 ITS fungal gene sequences were obtained from all the soil samples, encompassing 4,248 bacterial and 276 fungal OTUs.

**FIGURE 2:**
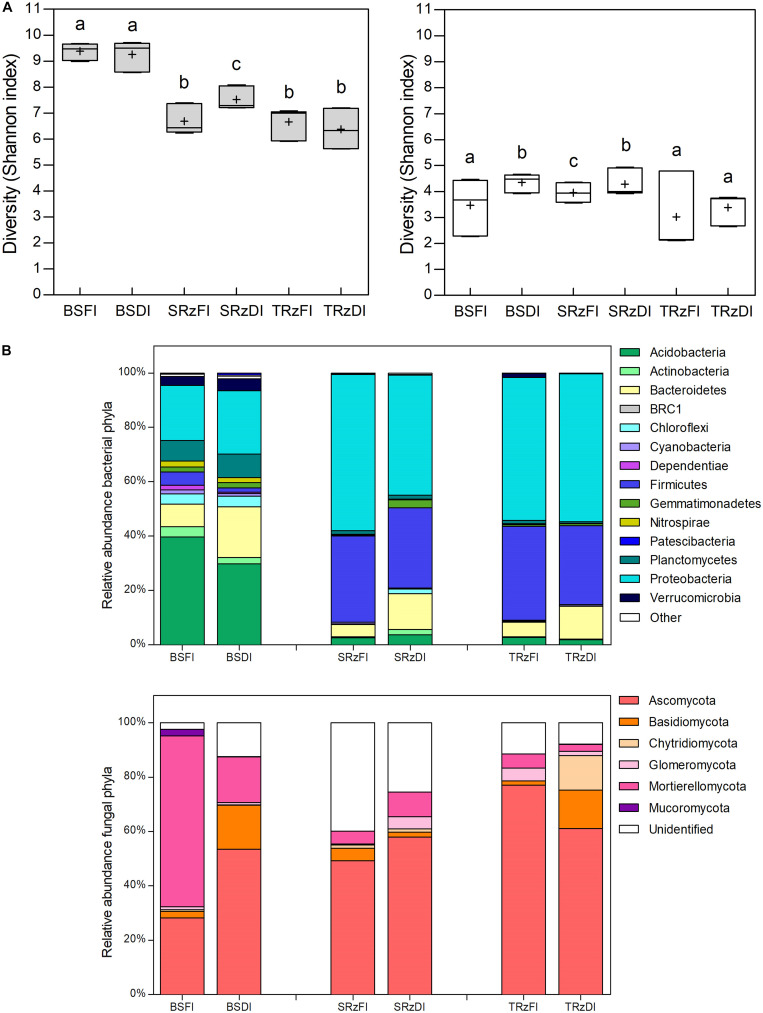
Diversity and taxonomic composition of soil microbial communities. **(A)** Ten sub samplings of random sequences without replacement of each sample were used to compare the diversity Shannon index between the soil type/water irrigation treatments (*n* = 30; gray bars bacteria; white bars fungi). Horizontal bars within boxes represent the median; crosses represent the media. The tops and bottoms of the boxes represent 75th and 25th quartiles, respectively. Bars with different letters indicate statistically significant differences (Kruskal–Wallis analysis of variance *p* < 0.05 and Dunn’s *post hoc* test). **(B)** Average phylum taxonomic composition of bacterial (upper panel) and fungal (lower panel) communities from different water irrigated soils (*n* = 3). Different colors represent distinct phyla. Other represent phyla with relative abundances < 1% in all samples. FI, full irrigation; DI, deficit irrigation. BS, bulk soil; SRz, susceptible cultivar rhizosphere; TRz, tolerant cultivar rhizosphere.

Microbial alpha diversity metrics (Figure 2 and Supplementary Figure 3) showed that the highest bacterial diversity (Shannon index) values belonged to the BS samples (Figure 2A, gray bars). Furthermore, a significant bacterial diversity increase was observed in the SRz comparing DI with FI, while the BS and TRz did not show differences between treatments. Similarly, the diversity of fungal communities of the SRz showed an increase in DI compared with FI, while the TRz samples were not significantly different between treatments (Figure 2A, white bars). On the other hand, microbial beta diversity analyses at the phylum level (PCoA and UPGMA) revealed that the bacterial communities showed clear segregation between the rhizosphere and BS samples irrespective of their irrigation treatment (Supplementary Figures 4A,B), while at the family level, only one SRzDI sample clustered together with the BS samples (Supplementary Figures 4C,D). This separation between BS and rhizospheres was not observed in the fungal communities (Supplementary Figure 5); yet they displayed a notable segregation of the fully irrigated bulk soils with respect to the other samples at both the phylum and family levels.

To assess the effect of plants on the soil microbial response to water deficit, we first compared the microbiome of both rhizospheres with that of the BS. Then, we compared the microbiome of the TRz and SRz to determine the effect of plant phenotype on the microbiome response to water deficit. Regarding the bacterial response to water deficit (Supplementary Table 2), at the phylum taxonomic level, Planctomycetes and Patescibacteria significantly increased their abundance (were over-represented) in DI compared with FI in the BS samples (Figure 2B, upper panel). However, no phylum significantly changed their abundance in the rhizosphere samples. At the order level, Chitinophagales, Clostridiales, and Candidatus Kaiserbacteria were over-represented in the BS samples under DI. In the SRz, Caulobacterales, RCP2-54, Blastocatellales, and Bdellovibrionales significantly increased their abundance in response to DI, while in the TRz, Solirubrobacterales and Chthoniobacterales did so. Interestingly, no common over-represented orders were found between the rhizospheres and BS, or between the rhizospheres of susceptible and tolerant cultivars to water deficit. Finally, at the OTU level, 84, 76, and 22 OTUs were over-represented in response to DI in the BS, SRz, and TRz, respectively. Remarkably, only two OTUs were shared between the rhizospheres and BS (both belonging only to SRz), and there were no common OTUs between TRz and SRz. Consequently, the 22 OTUs over-represented in TRz were exclusive to this cultivar rhizosphere in response to DI, which belong to the genera *Erythrobacter*, *Hydrocarboniphaga*, *Rhodoplanes*, *Sarcina*, *Solirubrobacter*, *JGI 0001001-H03*, *RB41*, *Altererythrobacter*, *Microvirga*, *Ellin6067*, *Candidatus Berkiella*, *Steroidobacter*, *Pseudomonas*, and *Bacillus*. Regarding the fungal response to water deficit (Supplementary Table 3), no phylum significantly increased its abundance in DI compared with FI in the rhizospheres or in the BS samples (Figure 2B, lower panel). At the order level, Cantharellales, Pleosporales, and Glomerellales were over-represented in BS under DI, while in the rhizospheres, no order significantly changed its abundance in DI compared with FI. Finally, at the OTU level, 14, 2, and 13 OTUs were over-represented in response to water scarcity in BS, SRz, and TRz, respectively, with no OTU shared among soils. Thus, the 13 OTUs over-represented in TRz that belong to the genera *Alternaria*, *Thanatephorus*, *Cercospora*, *Cryptococcus*, *Mycosphaerella*, *Mortierella*, *Penicillium*, and *Capronia* were exclusive to the tolerant cultivar in response to DI. Taken as a whole, the results suggest that these bacterial and fungal taxa that respond to DI exclusively in TRz could have an impact on the adequate adaptation of tomatoes to water scarcity.

### Interaction Network Analysis of Soil Microbial Communities Established Under Water Deficit

**FIGURE 3:**
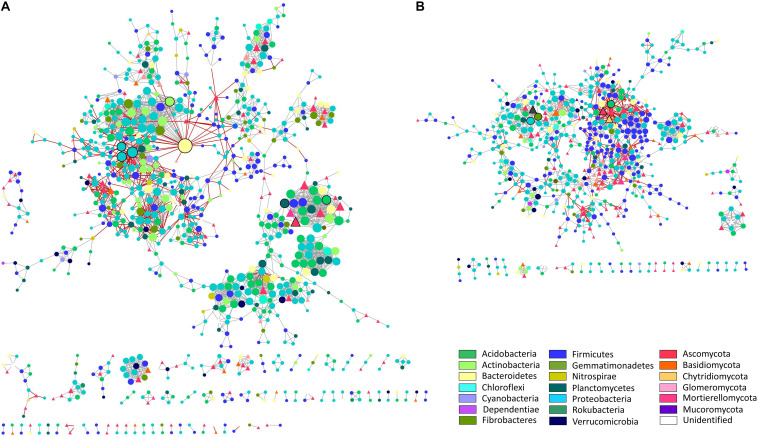
Microbial interaction networks from susceptible and tolerant cultivar rhizospheres. **(A)** Susceptible cultivar rhizosphere bacterial and fungal interaction network in response to water deficit. **(B)** Tolerant cultivar rhizosphere bacterial and fungal interaction network in response to water deficit. Interactions were inferred from a global microbial operational taxonomic unit (OTU) ratio (deficit irrigated over full irrigated plants) abundance. Each node represents an OTU, and each edge represents a significant pairwise association between them (gray lines: positive co-response to water deficit; red lines: negative co-response to water deficit). Nodes in the shape of circles are bacteria, and nodes in the shape of triangles are fungi. Different colors of nodes represent distinct phyla. Node size is proportional to the number of connections (degree) for both networks (maximum node degree for SRz network is 39 and for TRz network is 20). Nodes with black border represent “responsive-to-water-deficit hub nodes” (1% of OTUs with the highest degrees in each network; eight SRz hub nodes; six TRz hub nodes).

To further analyze the rhizosphere microbiomes associated with differential responses of the tolerant and susceptible plants to water deficit, we unraveled interactions of the integrated bacterial and fungal communities through network analysis (Figure 3 and Supplementary Figure 6). This analysis only recovered the microbial interactions established entirely from the water deficit response at each soil. Thus, while the nodes of the networks represent OTUs annotated at the phylum level, the edges represent positive or negative abundance correlations between nodes in response to water deficit. The results showed that network metrics were different between soil compartments (Table 1). For instance, the BS network possessed 294 connected components, followed by 67 and 36 connected components in the SRz and TRz networks, respectively, showing less segregation in the TRz. Furthermore, the BS network also contained the highest number of nodes and edges (1,940 nodes and 6,484 edges), while the rhizosphere networks were smaller, comprising 891 nodes and 2,838 edges in SRz, and 648 nodes and 1,651 edges in the TRz network. These differences among the networks were also observed at the taxonomic level of the nodes. While the BS network displayed 32 identified bacterial and fungal phyla, the SRz and TRz networks contained nodes belonging to 25 and 23 phyla, respectively (Supplementary Table 4). Thus, these results indicate that the rhizosphere networks, especially the TRz network, are more compact and less complex than the BS network. Despite these differences, the bacterial phyla Verrucomicrobia, Planctomycetes, Gemmatimonadetes, and Actinobacteria, but no fungal phyla, were over-represented in all three interaction networks (BS, SRz, and TRz), compared with their relative abundance in their respective communities (Supplementary Tables 2, 3). This suggests that these bacterial taxa may be relevant in the microbial interactions established in response to water deficit, regardless of their low relative abundance in the microbial communities (between 0.2 and 8.1% average relative abundance).

**TABLE 1:** Parameters of microbial interaction networks.

Parameter	BS	SRz	TRz
Number of connected components	294	67	36
Number of nodes	1,940	891	648
Bacterial nodes	1,818	770	533
Fungal nodes	122	121	115
Number of total positive interactions	5,426	2,359	1,376
Number of total negative interactions	1,058	479	275
Total ^+/–^ interaction ratio	*5.13*	*4.92*	*5.00*
Number of bacterial positive interactions	4,883	1,861	947
Number of bacterial negative interactions	846	396	134
Bacterial ^+/–^ interaction ratio	*5.77*	*4.70*	*7.07*
Number of fungal positive interactions	19	58	81
Number of fungal negative interactions	9	11	3
Fungal ^+/–^ interaction ratio	*2.11*	*5.27*	*27.00*
Number of bacteria-fungi positive interactions	524	440	348
Number of bacteria-fungi negative interactions	203	72	138
Bacterial-fungal ^+/–^ interaction ratio	*2.58*	*6.11*	*2.52*

*In italic, the ^+/–^ interaction ratios.*

To deepen the network analysis, we identified “keystone” taxa (hyperconnected hub nodes) (Figure 3, Supplementary Figure 6, and Supplementary Table 4), which are critical for network assembly by displaying most co-responses to water deficit. In the BS network, we identified four fungi and 55 bacterial responsive-to-water-deficit hub nodes distributed in two of its 294 connected components. The SRz network showed only one fungus and seven bacterial hub nodes, while the TRz network showed three fungi and three bacterial hub nodes, with no responder hub nodes shared among the three networks. Remarkably, the node with the highest degree in the microbial networks (*n* = 39 interactions) belonged to the SRz and was the only Bacteroidetes responsive-to-water-deficit hub node identified in the rhizosphere networks, indicating a specific and relevant role of this Microscillaceae family member in the response of the SRz to water deficit.

On the other hand, regarding the total network community ratio of positive over negative links, we observed that these were similar between soils (5.13, 4.92, and 5 for the BS, SRz, and TRz networks). However, bearing solely bacterial interactions, the BS network had a positive/negative ratio of 5.77, while SRz and TRz ratios were 4.7 and 7.07, respectively (Table 1). Involving only fungal interactions, the BS network had the lowest positive/negative ratio (2.11), followed by the SRz ratio (5.27), and, finally, highlighted the high positive/negative fungal ratio in the TRz network (27.00). Lastly, the positive/negative ratio between bacterial and fungal interactions corresponded to 2.58 in the BS network and 6.11 in the SRz, while the lowest ratio belonged to the TRz network (2.52). Hence, the network analysis indicated that the water deficit-tolerant phenotype in tomatoes correlated with a higher incidence of positive intra-kingdom and negative inter-kingdom interactions between members of the rhizosphere microbiome.

## Discussion

In this study, we systematically selected *S. lycopersicum* cultivars tolerant and susceptible to water deficit based on the physiological and morphological response of 72 tomato cultivars to low water availability. For the final selection, we measured RWC, Pn, and Ψ_stem_ as sensitive water stress indicative traits (Cooper et al., 2003; Rampino et al., 2006; Skillman et al., 2011; Poot and Veneklaas, 2013; Reinhardt et al., 2015; Fernandes-Silva et al., 2016; Wang et al., 2018; Supplementary Table 1), considering as the most susceptible (ADV) or tolerant (449) those cultivars that reduced or did not change these physiological parameters in response to water deficit, respectively (Figure 1). Remarkably, we observed that only the susceptible cultivar displayed a significant decrease in root areas in DI compared to FI (Supplementary Figure 2), in concordance with other studies performed on susceptible plants submitted to drought (Xiong et al., 2006; Brunner et al., 2015). Therefore, since the root of tolerant and susceptible tomato cultivars responded to the water deficit condition differently, we evaluated the root-associated bacterial and fungal rhizosphere microbiomes of ADV and 449 tomato cultivars to water deficit by HTS.

Considering all soils, we identified more bacterial (4,248) than fungal (276) OTUs in the soil and plant-associated microbiota, as has been reported in other studies (Coller et al., 2019; Trivedi et al., 2020). Due to this numerical imbalance, changes in the abundance of any fungus could be relevant to the community structure; thus, the abundance of members should be incorporated in the analyses, reason why diversity was preferred over richness in this study. The results indicated that bacterial diversity was higher in the BS samples than in the rhizosphere microbiomes (Figure 2A, upper panel), a result that agrees with other studies that have also observed diminished bacterial alpha diversity parameters within soils from the root zone when compared with plant free soils (Shi et al., 2011; Bulgarelli et al., 2012; García-Salamanca et al., 2013; Li et al., 2016; Qiao et al., 2017; Fernández-Gómez et al., 2019). This plant effect was also observed in the PCoA and UPGMA tree of the bacterial but, interestingly, not in the fungal communities (Supplementary Figure 4), which suggests an effect of root exudates specifically on the bacterial soil community, for instance, by the release of antibacterial metabolites (Bais et al., 2002, 2005). Concerning the irrigation treatments, the analysis showed that under FI, the fungal but not the bacterial community diversity was sensitive to the type of cultivar. However, under DI, both bacterial and fungal community diversities increased in the SRz but not in the TRz (Figure 2A), indicating that the tolerant cultivar exerts a buffering effect on bacterial and fungal communities in face of water deficit. This not only implies that there is a close link between plant variety and the rhizosphere microbial community, as has already been described elsewhere (Berg and Smalla, 2009; Bouffaud et al., 2014; Ofek-Lalzar et al., 2014; Bulgarelli et al., 2015; Li et al., 2018), but that this host-microbial differentiation seems to be associated to the divergent capacities of the plants to adapt to water scarcity. Nevertheless, more studies that include additional water deficit differentially tolerant cultivars are needed to evaluate this assessment.

When deepening in the microbiomes of the soils, different taxa were specific for each soil type and irrigation treatment (Supplementary Tables 2, 3). In the case of bacteria, the order Solirubrobacterales (phylum Actinobacteria) and OTUs belonging to this taxon were over-represented in the TRz samples under DI, suggesting a special role of these microorganisms in the adaptation of tomato tolerance to water scarcity. Notably, several studies have shown that Actinobacteria are agriculturally crucial, as they can enhance plant vigor and confer tolerance to abiotic stresses, such as drought (Franco-Correa and Chavarro-Anzola, 2016), within which specifically Solirubrobacterales were shown to be beneficial microorganisms for plant growth promotion (Franke-Whittle et al., 2015).

Moreover, some of the fungal over-represented OTUs in the TRz under DI also belonged to genera that have been identified as beneficial for plant growth, such as *Mortierella* and *Penicillium* (Murali et al., 2013; Borrell et al., 2017; Gómez-Muñoz et al., 2017). Thus, these specific microorganisms from the TRz under DI that belong to taxa with known beneficial characteristics for plants, could be contributing to the tolerant phenotype, highlighting the metabolic bidirectionality of the holobiont system. Remarkably, some other fungal over-represented OTUs in the TRz under DI have also been associated with plant pathogenesis (Zhang et al., 2005; Nadziakiewicz et al., 2018; Campos-Rivero et al., 2019; Jastrzêbska et al., 2020). Notwithstanding, since none of the plants in this study showed signs of any disease, these results emphasize that the development of an infectious disease depends on particular environmental factors and host-microbe interactions (Bass et al., 2019), being some potential pathogens even plausibly relevant for the adaptation of plants to water deficit.

An important aspect to consider regarding taxa identification by HTS is primer selection. Combinations of bacterial and fungal primers have shown different types of biases regarding the relative abundance of some community members (Tedersoo et al., 2015; Thijs et al., 2017). For instance, taxa falling into the arbuscular mycorrhizal group of Glomeromycota, which are typical fungal phyla that allow host plants to grow more efficiently under biotic and abiotic stress conditions (Gholamhoseini et al., 2013; Jayne and Quigley, 2014), have shown disproportionate OTU abundances using ITS2 primer (Tedersoo et al., 2015). Although in this study we did not find significant differences in this phylum comparing the irrigation treatments in the different soil samples, the selection of microbial primers for HTS is a relevant point to address in order to achieve precision in relative abundance analysis trials.

To further understand how the differential response of tomato plants to water deficit affects the microbial community beyond their changes in relative abundance, in this study we established a new approach that involved the use of the abundance ratios obtained in DI with respect to FI to generate microbial interaction networks. This approach offers new insights into the microbial ecological rules that guide community assembly in the face of a condition of interest. One of the most noteworthy results from the network interaction analysis was the difference between the complexity of the networks. For instance, the TRz network showed the lowest number of connected components, nodes, and edges in response to water deficit (Table 1); hence, the TRz microbiota displayed the smallest and least complex soil network. Thus, although water deficit did not affect the microbial diversity of the tolerant cultivar, it could be affecting the interactions established among the members of its community. In fact, the data showed that in the TRz network, the intra-kingdom ratio of positive over negative links was considerably higher than in the other networks, while the inter-kingdom ratio was the lowest (Table 1).

Since the surplus of positive over negative abundance pattern correlations is assumed as an engagement in a cooperative metabolism that could provide health benefits for the host (Bäckhed et al., 2005; Eberl, 2010; Sleator, 2010; Van den Abbeele et al., 2011), mainly because the competition between microbes (negative links) has the potential to severely reduce the efficiency of any cooperative metabolism that benefits the host (Bäckhed et al., 2005; Eberl, 2010; Sleator, 2010; Van den Abbeele et al., 2011), we hypothesize that the reduction in the TRz network resulted in the microbial community having to become more efficient, which is reflected in increased cooperation within kingdoms. In addition, less cooperation between kingdoms can be explained by optimization of resources that reasonably tends to favor members of the same kingdom, based on the concept that antagonism between bacteria and fungi is connected to competition for substrate (Mille-Lindblom et al., 2006).

Remarkably, the “keystone” taxa that have the most considerable influence in the different communities (Vick-Majors et al., 2014; Banerjee et al., 2016) from the BS and SRz networks, where highly predominated by bacteria, while in the TRz network, hub nodes belonged equally to bacteria and fungi (Supplementary Table 4). Thus, in the case of the tolerant rhizosphere, members from both kingdoms sculpt the microbial assemblages and exert a strong influence on the community.

To the best knowledge of the authors, this is the first HTS microbial study that evaluated the taxonomic composition and interaction patterns of microbial communities from thoroughly designated tolerant or susceptible plant cultivars under water deficit. The results highlight the relevance of considering the holobiont (and not only one of its parts) to interpret plants’ adaptability to water deficit and suggests that its surrounding microbiota conditions the plant’s phenotype. New experimental approaches, such as exudate metabolomics, could be incorporated in future analyses to demonstrate the correlation between differential root exudates and the distinct rhizosphere microbial communities between tolerant and susceptible plants to low water availability. Additionally, assays involving the exchange of rhizospheres between drought-tolerant and susceptible cultivars could elucidate if the entire microbial communities are functional in contributing to the tolerance of plants to water deficit.

## Data Availability Statement

The datasets presented in this study can be found in online repositories. The names of the repository/repositories and accession number(s) can be found below: https://www.ncbi.nlm.nih.gov/, PRJNA649642.

## Author Contributions

AG: methodology, validation, investigation, data curation, writing—original draft, and project administration. RP: conceptualization, methodology, investigation, resources, writing—original draft, project administration, and funding acquisition. CH and JM: validation, formal analysis, resources, data curation, and writing—review and editing. LP: methodology, validation, investigation, resources, writing—review and editing, and funding acquisition. DZ: writing—review and editing, supervision. CP: resources, writing—review and editing, and supervision. MG: conceptualization, resources, writing—review and editing, and funding acquisition. NF: conceptualization, methodology, resources, and supervision. DM: conceptualization, methodology, investigation, resources, data curation, writing—original draft, project administration, supervision, and funding acquisition. All authors contributed to the article and approved the submitted version.

## Conflict of Interest

The authors declare that the research was conducted in the absence of any commercial or financial relationships that could be construed as a potential conflict of interest.
