# Supplementary figures and images for: Tomato Cultivars With Variable Tolerances to Water Deficit Differentially Modulate the Composition and Interaction Patterns of Their Rhizosphere Microbial Communities
Source: Front Plant Sci. 2021 Jul 13;12:688533. doi: 10.3389/fpls.2021.688533 (PMC8313812; doi:10.3389/fpls.2021.688533)

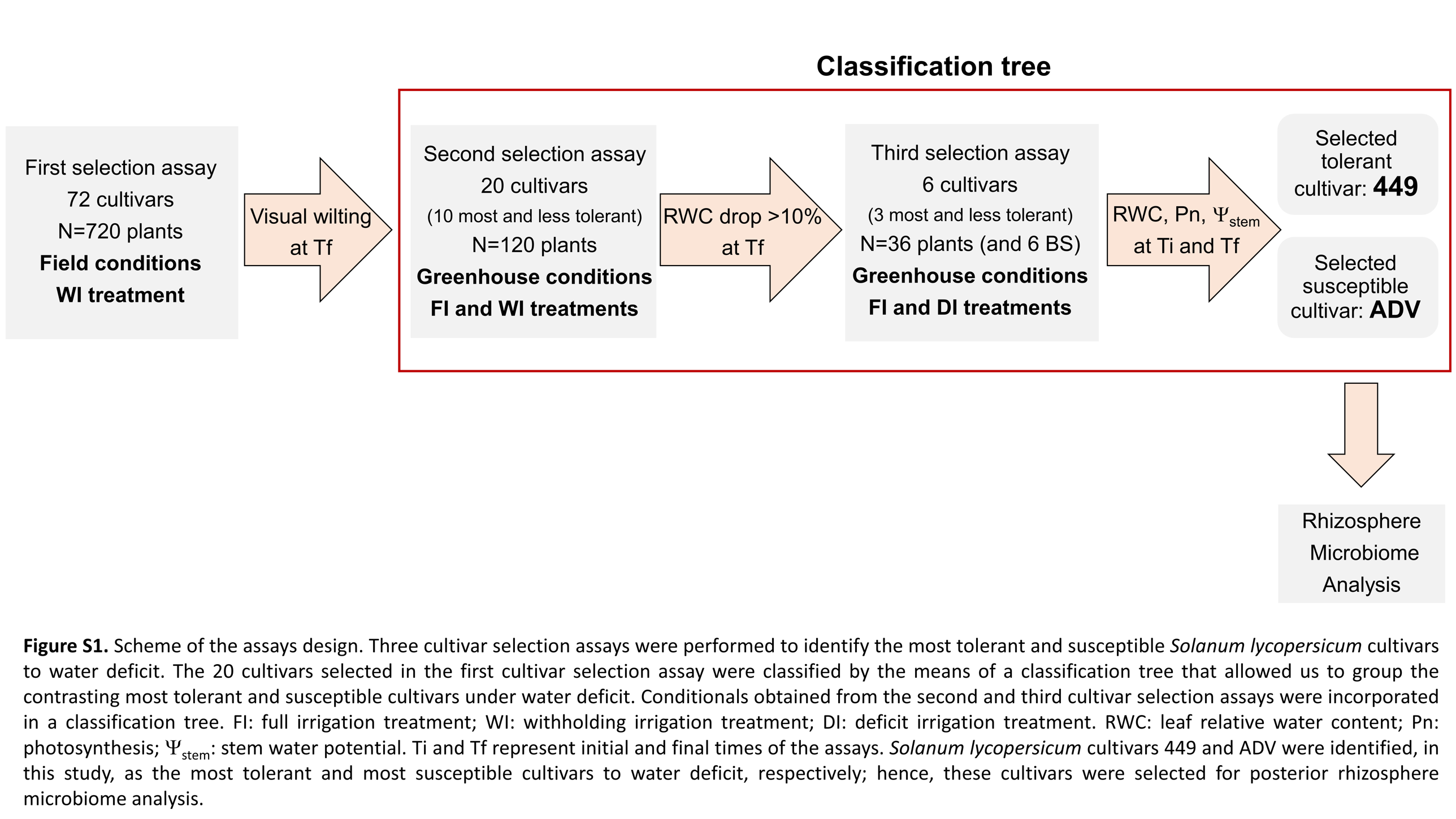

Supplement: Supplementary file 1 [file Image_1.JPEG]

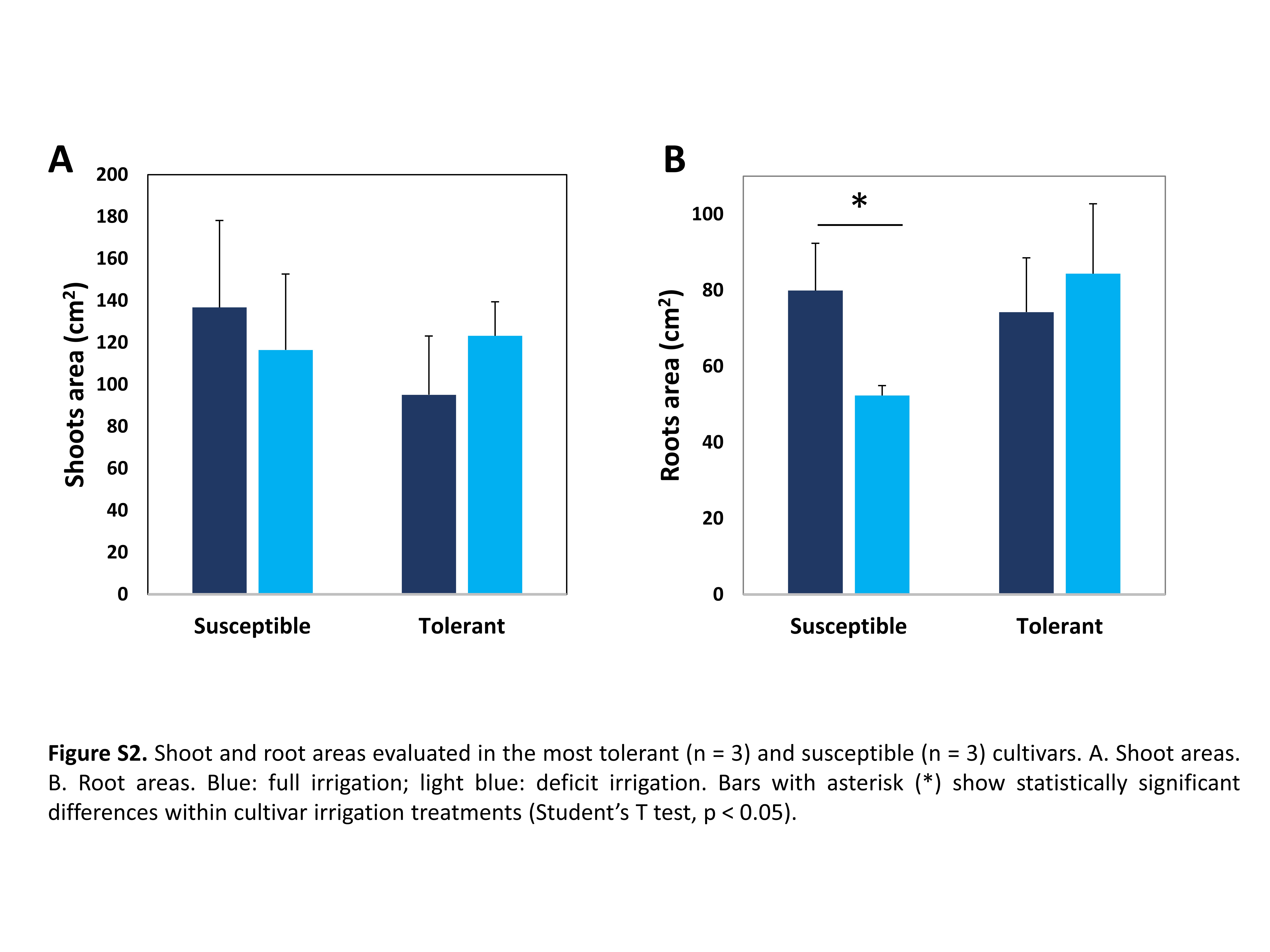

Supplement: Supplementary file 2 [file Image_2.JPEG]

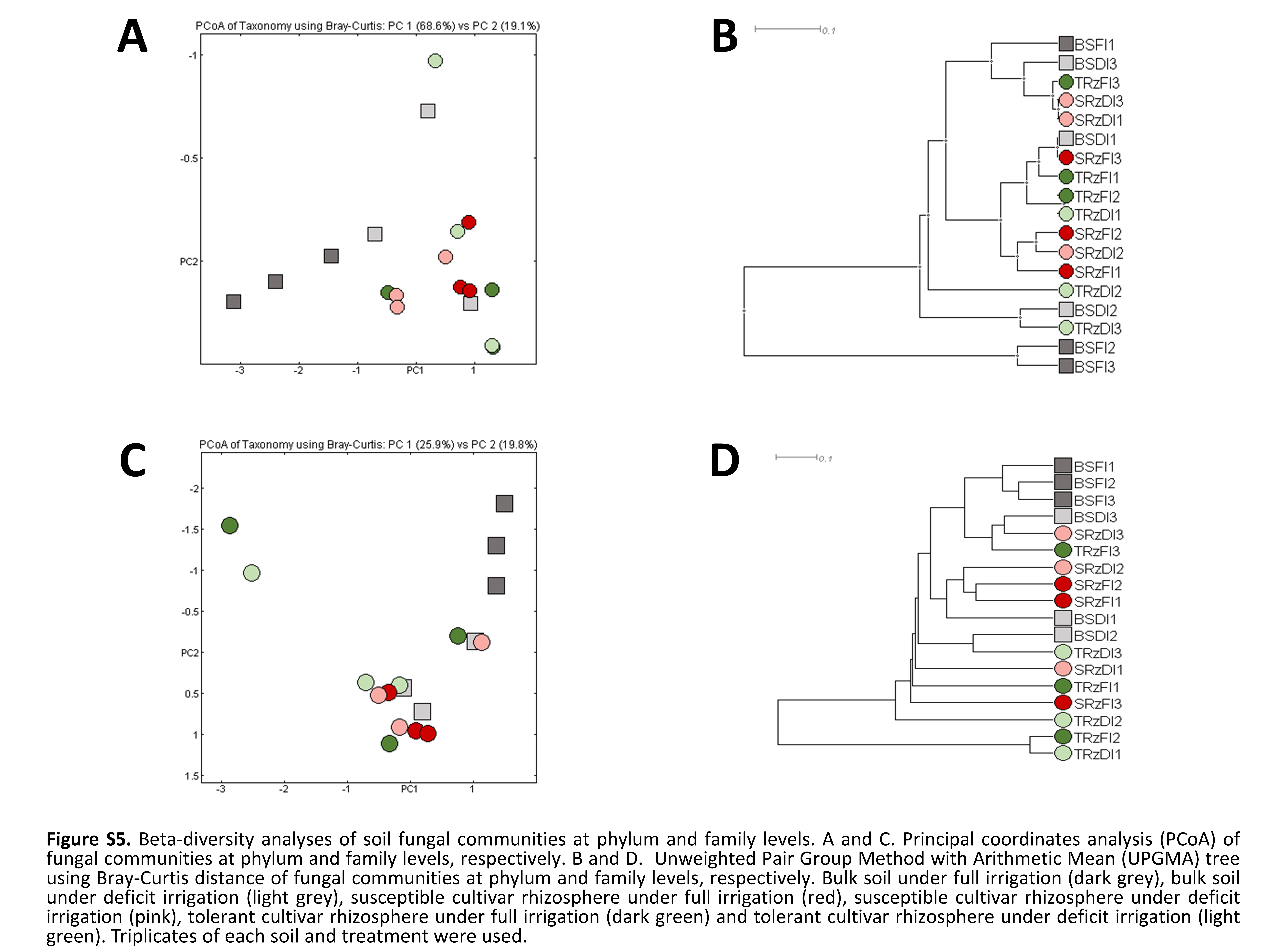

Supplement: Supplementary file 5 [file Image_5.JPEG]

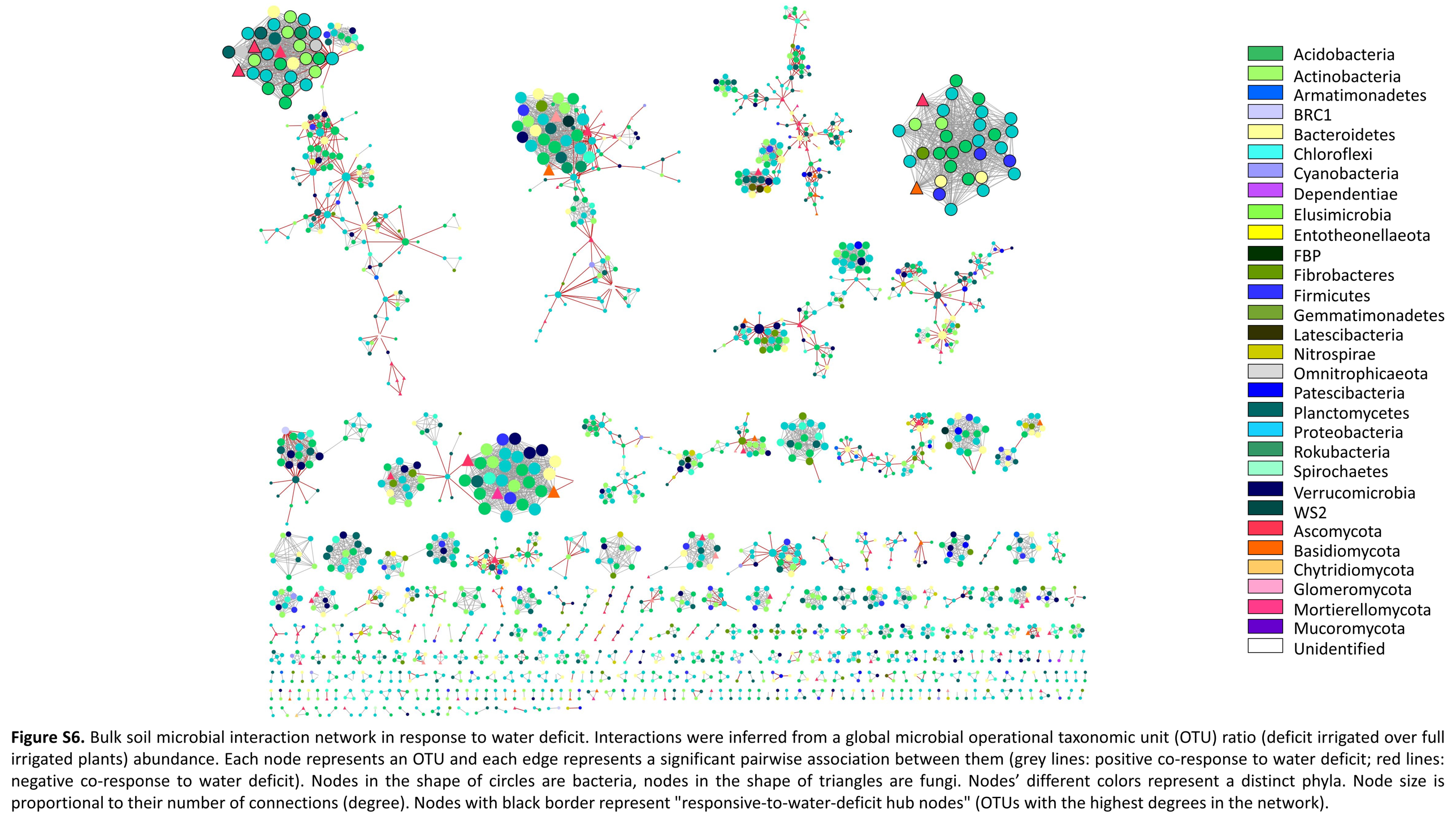

Supplement: Supplementary file 6 [file Image_6.JPEG]
